# Curve flexibility in cerebral palsy-related neuromuscular scoliosis: does the intraoperative prone radiograph reveal more flexibility than preoperative radiographs?

**DOI:** 10.1186/s13013-017-0122-2

**Published:** 2017-05-04

**Authors:** Zubair Chaudry, John T. Anderson

**Affiliations:** 1 Children’s Mercy – Kansas City, 2401 Gillham Rd, Kansas City, MO 64108 USA; 2 Department of Orthopaedic Surgery, University of Missouri-Kansas City School of Medicine, Kansas City, MO 64108 USA; 3 201 State St., Erie, PA 16550 USA

**Keywords:** Neuromuscular scoliosis, Spine flexibility, Cerebral palsy

## Abstract

**Background:**

Spinal flexibility is determined preoperatively by manipulating the spine and assessing, radiographically, to what extent the amount of deformity reduces. Quantifying spinal flexibility is important when determining the approach to the planned operation in order to achieve the most optimal spinal correction and balance. Currently, supine traction radiography is a popular method used in patients with severe, cerebral palsy-related neuromuscular scoliosis.

The different methods for determining spinal flexibility have been studied extensively in the adolescent idiopathic scoliosis population. No such studies exist in the cerebral palsy population. The purpose of this study was to determine how predictive the intraoperative prone radiograph is in determining spinal flexibility in patients with severe, cerebral palsy related neuromuscular scoliosis. ﻿ Furthermore, the intraoperative prone radiograph was compared to the preoperatively acquired supine and supine traction radiographs.

**Methods:**

Twenty-five consecutive patients with severe, cerebral palsy-related neuromuscular scoliosis were studied. The Cobb angles of the preoperative supine, preoperative supine traction, and intraoperative prone radiograph were measured and compared. The flexibility indices of these radiographs were calculated and compared.

Traction was not applied during acquisition of the intraoperative prone radiograph. The radiograph was taken during the exposure to localize surgical levels, prior to instrumentation.

**Results:**

The supine traction radiograph and the intraoperative prone radiograph had higher flexibility indices than the preoperative supine radiograph. These comparisons were statistically significant. The comparison between the flexibility indices of the supine traction radiograph and intraoperative prone radiograph was not statistically significant.

When looking at the preoperative supine traction radiograph separately, it was noted that the process of instrumentation led to 30% more correction of the Cobb angle.

**Conclusions:**

The intraoperative prone radiograph is more predictive of spinal flexibility in patients with severe scoliosis related to cerebral palsy when compared to the preoperative supine radiograph but not the preoperative supine traction radiograph. The preoperative supine traction radiograph serves as the optimal method for determining spinal flexibility in patients with severe, cerebral palsy-related neuromuscular scoliosis.

## Background

Surgical treatment for scoliosis aims to reduce the magnitude of the deformity, to obtain fusion, and to prevent future progression of the curve [1]. Preoperative spine flexibility is assessed in patients with scoliosis to predict the degree of correction that will result from instrumentation. In addition, spinal flexibility is often used to determine the surgical approach required to achieve maximum correction of the deformity. More traditional options include the posterior, the anterior, or the combined approach [2]. More rigid and larger curves may require the combined approach for the desired amount of correction, which has a higher complication rate and requires a longer operative time [3]. With more accurate assessment of curve flexibility preoperatively, patients with presumably more rigid and pronounced curves could be spared from undergoing an anterior procedure and thus avoid its inherent risks [4].

Klepps et al, Davis et al, Lui et al, and Hamzaoglu et al, have compared several methods for assessing preoperative flexibility in patients with adolescent idiopathic scoliosis (AIS). These methods included traction radiography under general anesthesia, push prone radiographs, and supine side-bending radiographs [3–6]. Although radiographic methods for assessing curve flexibility have been studied in the AIS population, little information exists regarding various methods in patients with cerebral palsy.

Supine antero-posterior (AP) radiograph performed during the application of longitudinal traction is a common method for determining spinal flexibility in patients with cerebral palsy [2]. Due to their dystonia and cognitive delay, it is often difficult or impossible for children with cerebral palsy to participate in more traditional means of assessing spinal flexibility such as supine side-bending radiographs.

As the curves in cerebral palsy patients tend to be larger and more rigid, the surgeon is often left with the decision of whether an anterior-posterior procedure will be necessary to achieve proper spinal balance [7]. It has been the principal investigator’s impression that curve flexibility is often underestimated on preoperative radiographs when compared to the intraoperative prone radiograph obtained during exposure, while under general anesthesia, to localize surgical levels. The purpose of this study was to assess how the intraoperative prone radiograph compares the preoperative supine and preoperative supine traction radiographs for assessing spinal flexibility.

## Methods

Twenty-five consecutive patients with severe, cerebral palsy-related neuromuscular scoliosis who underwent posterior-instrumented spinal fusion were studied retrospectively. Our Institutional Review Board approved the protocol for the study.

All 25 patients carried the diagnosis of cerebral palsy (GMFCS 5). Patients with other diagnoses were excluded. Patients with a history of previous spinal operations were excluded. Patients were also excluded if they were younger than 10 years or older than 21 years.

Cobb angles were measured and recorded from the following patient radiographs: preoperative sitting AP radiographs, preoperative supine radiographs, preoperative supine traction radiographs, intraoperative prone radiographs, and the first postoperative sitting AP radiograph.

Traction was not applied during acquisition of the intraoperative prone radiograph. The radiograph was taken during the exposure to localize surgical levels, prior to instrumentation.

Flexibility indices were calculated for the radiographs utilizing the following formula:

- Flexibility index = (preoperative Cobb angle − corrected Cobb angle)/(preoperative Cobb angle)
- In addition to the flexibility indices, the correction rate was also determined. The correction rate determines the degree of spinal correction obtained by the operation compared to the initial preoperative sitting Cobb angle. The following formula was utilized:
- Correction rate = (preoperative sitting Cobb angle − postoperative sitting Cobb angle)/(preoperative sitting Cobb angle)

### Data analysis

We summarized the outcome variables (flexibility indices and correction rates) using means and standard deviations. We compared the flexibility indices among the different types of radiographs using paired *t* tests. The correction rates were also compared to the flexibility indices in the same manner. Statistical software was SPSS 2.0.

## Results

There were 25 patients total. The average age of the patients studied was 13.1 (range 10–19). The mean curve measurement on the preoperative sitting AP radiograph was 91°(range 70°–115°), preoperative supine was 68°(range 22°–95°), preoperative supine traction was 54°(range 16**°**–78**°**), and intraoperative prone was 55° (range 28°–75°). The average flexibility index for the preoperative supine radiograph was 25%, the preoperative traction radiograph was 41%, and the intraoperative prone radiograph was 40%. The average correction rate was 70%.

### Flexibility indices

Table 1 lists the average flexibility indices and percent correction for each of the studied radiographs. Table 2 lists the comparisons of the flexibility indices among the different radiographs. Both the preoperative supine traction radiograph and the intraoperative prone radiograph showed significantly superior flexibility indices compared to the preoperative supine radiograph (*p* < 0.001 for both comparisons). When comparing the flexibility indices of the preoperative supine traction radiograph directly with the intraoperative prone radiograph, there was no statistically significant difference (*p* = 0.308).

**Table 1:** Average flexibility indices for the studied radiographs and percent correction for all subjects

	Supine FI	Traction FI	Intraop. FI	% Correction
Mean (%)	25.28	41.36	39.92	70.2
SD	14.953	16.395	14.836	10.316
*N*	25	25	25	25

*FI* flexibility index, *SD* standard deviation, *N* number of patients

**Table 2:** Comparisons of the flexibility indices among the different radiographs

Radiographs	Mean	SD	95% CI of the difference		*p* value
			Lower	Upper	
Supine FI–Traction FI	−16.1	7.7	−19.2	−12.9	<0.001
Supine FI–Intraop. FI	−14.6	8.9	−18.3	−11	<0.001
Traction FI–Intraop. FI	1.4	6.9	−1.4	4.3	0.308

*FI* flexibility index, *SD* standard deviation, *CI* confidence interval

When looking at the preoperative supine traction radiograph separately, it was noted that the process of instrumentation led to 30% more correction of the Cobb angle.

## Discussion

In the AIS population, supine side-bending radiographs are utilized routinely for determining spinal flexibility. Radiographs obtained after applying longitudinal traction with the patient supine serve as an optional method for patients with more pronounced curves and those that are uncooperative [5]. Patients with severe cerebral palsy, like the patients of this study, are unable to participate in side-bending radiographs and have stiffer and larger curves. Thus, traction radiography is a popular method used among this population.

In the AIS population, there have been numerous studies that have compared various radiographic techniques in determining their propensity for predicting the amount of surgical correction attainable [3–6]. To our knowledge, no such studies exist for the cerebral palsy population.

The purpose of this study was to determine the value of the intraoperative prone radiograph in determining spinal flexibility. We hypothesized that it provided more accurate information compared to our standard preoperative radiographs. However, when comparing the preoperative supine traction radiograph to the intraoperative prone radiograph, there was no statistical significance between the flexibility indices of the two radiographs.

The data from our study revealed that the preoperative supine traction radiograph is the most useful for determining preoperative spinal flexibility in the severe cerebral palsy population. The intraoperative radiograph is not more predictive of spinal flexibility compared to the traction radiograph. Furthermore, from a resource utilization and patient safety standpoint, information gathered preoperatively and without the need for an additional anesthetic is more pragmatic. Furthermore, we felt the data might help surgeons predict, to some extent, the amount of correction that is obtainable by means of a posterior procedure utilizing pedicle screw instrumentation. When looking at the preoperative supine traction radiograph separately, it was noted that the process of instrumentation led to 30% more correction of the Cobb angle. This information might help surgeons plan their surgical approach.

Although the number of patients in this study is relatively small, the data collected is statistically significant. In order to reveal a 10% difference between the flexibility indices of the supine traction view and the intraoperative prone view, 26 patients would be needed, assuming a standard deviation of 18, to have 80% power. Because our standard deviation was lower, we felt 25 patients were adequate for the purposes of this study. Obviously, hundreds of patients would be necessary to detect a significant difference between the two radiographs, as the actual difference between their flexibility indices is quite small.

## Conclusions

The intraoperative prone radiograph is more predictive of spinal flexibility in patients with severe scoliosis secondary to cerebral palsy than the preoperative supine radiograph but not the preoperative supine traction radiograph. Overall, the supine traction radiograph serves as the optimal method for determining spinal flexibility in patients with severe, cerebral palsy-related neuromuscular scoliosis. The preoperative supine traction radiograph may help surgeons predict the amount of correction to be anticipated.

## Abbreviations

AIS: Adolescent idiopathic scoliosis
AP: Antero-posterior
GMFCS: Gross motor functional classification system
PA: Postero-anterior
